# The Inhibition of Anti-DNA Binding to DNA by Nucleic Acid Binding Polymers

**DOI:** 10.1371/journal.pone.0040862

**Published:** 2012-07-11

**Authors:** Nancy A. Stearns, Jaewoo Lee, Kam W. Leong, Bruce A. Sullenger, David S. Pisetsky

**Affiliations:** 1 Duke University Medical Center, Department of Medicine, Durham, North Carolina, United States of America; 2 Medical Research Service, Durham Veterans Administration Medical Center, Durham, North Carolina, United States of America; 3 Duke Translational Research Institute, Durham, North Carolina, United States of America; 4 Duke University Medical Center, Department of Surgery, Durham, North Carolina, United States of America; 5 Duke University, Department of Biomedical Engineering, Durham, North Carolina, United States of America; Beth Israel Deaconess Medical Center, United States of America

## Abstract

Antibodies to DNA (anti-DNA) are the serological hallmark of systemic lupus erythematosus (SLE) and can mediate disease pathogenesis by the formation of immune complexes. Since blocking immune complex formation can attenuate disease manifestations, the effects of nucleic acid binding polymers (NABPs) on anti-DNA binding *in vitro* were investigated. The compounds tested included polyamidoamine dendrimer, 1,4-diaminobutane core, generation 3.0 (PAMAM-G3), hexadimethrine bromide, and a β-cylodextrin-containing polycation. As shown with plasma from patients with SLE, NABPs can inhibit anti-DNA antibody binding in ELISA assays. The inhibition was specific since the NABPs did not affect binding to tetanus toxoid or the Sm protein, another lupus autoantigen. Furthermore, the polymers could displace antibody from preformed complexes. Together, these results indicate that NABPs can inhibit the formation of immune complexes and may represent a new approach to treatment.

## Introduction

Antibodies to DNA (anti-DNA) are the serological hallmark of systemic lupus erythematosus (SLE), a prototypic autoimmune disease characterized by the production of antibodies to components of the cell nucleus (antinuclear antibodies or ANA) in association with diverse clinical manifestations [1], [2]. Among these ANA, anti-DNA antibodies serve as markers for diagnosis and prognosis and play an important role in immunopathogenesis via the formation of immune complexes [3]–[5]. Thus, complexes of DNA and anti-DNA can deposit in the kidney to incite glomerulonephritis as well as induce the expression of type 1 interferon by plasmacytoid dendritic cells [6]–[8]. Cytokine induction depends on the stimulation of toll-like receptor (TLR) and non-TLR nucleic acid sensors, with antibodies promoting DNA internalization. Together, these findings have focused attention on anti-DNA antibodies as a target of therapy by inhibiting their production as well as their interaction with DNA [9]–[11].

At present, therapy for SLE involves non-specific immunomodulatory agents that, while frequently effective, have many side effects, including serious infection from immunosuppression [12], [13]. In view of the important role of anti-DNA in disease pathogenesis, investigators have explored more selective approaches to block the production of these antibodies or reduce their consequences [14]–[20]. Among these approaches, agents inhibiting the interaction of DNA and anti-DNA can prevent the formation of pathogenic complexes that deposit in the kidney or drive cytokine production. While oligonucleotides, peptides and small molecules can interact with antibody combining sites to block DNA interactions, such approaches can be limited by the heterogeneity of the anti-DNA response and the expression of antibodies that interact with diverse antigenic sites on the DNA molecule [4].

As a new approach for blocking immune complex formation, we have therefore explored the effects of agents that can interact with DNA as opposed to anti-DNA antibodies. For this purpose, we have investigated compounds termed nucleic acid binding polymers (NABPs). NABPs span a wide range of chemical structures and have been investigated primarily as agents to condense DNA into nanocomplexes that can be internalized by cells for nonviral gene therapy [21]. In the studies presented herein, we have tested three representative NABPs called PAMAM-G3 (polyamidoamine dendrimer, 1,4-diaminobutane core, generation 3.0), HDMBr (hexadimethrine bromide) and CDP (a β-cylodextrin-containing polycation). These compounds were studied in view of previous work indicating their ability to bind nucleic acids in blood [22], [23]. As results of these experiments show, NABPs can effectively inhibit the interaction of anti-DNA antibodies with DNA and even dissociate pre-formed immune complexes. These studies thus identify a new platform for developing inhibitors of anti-DNA activity that can selectively block autoantibody interactions that are key to the pathogenesis of SLE.

## Results

### Inhibition of Monoclonal Anti-DNA Binding by NABPs

**Figure 1:**
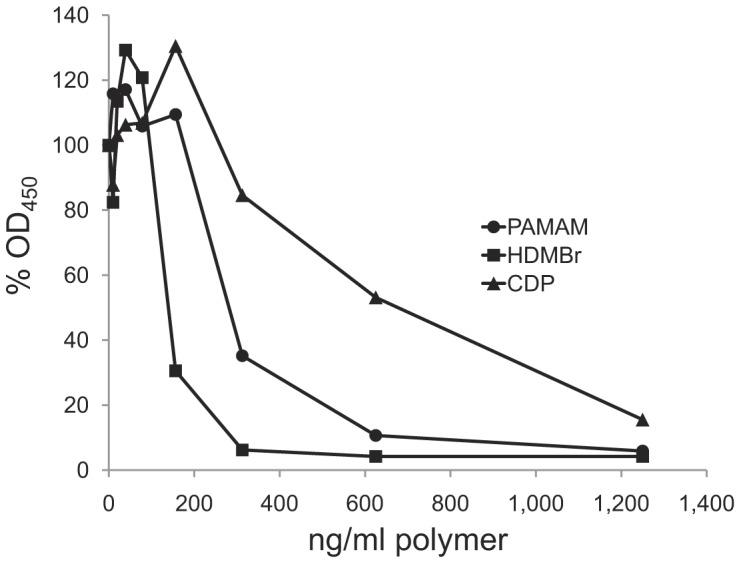
The inhibition of murine monoclonal anti-DNA binding to DNA by NABPs. The ability of polymers PAMAM, HDMBr, and CDP to inhibit binding of a murine monoclonal anti-DNA antibody QB1 to DNA was tested by ELISA. Binding of QB1 at 35 ng/ml final concentration was assayed by ELISA in the presence of PAMAM, HDMBr, or CDP at final concentrations from 10 ng/ml to 1,250 ng/ml. Control wells had buffer alone. Antibody was measured as described in Experimental Procedures. The OD_450_ values of 3 wells of each polymer concentration or of 6 wells without polymer were averaged. Each point shown is 100% x average OD_450_ with polymer/average OD_450_ without polymer. Circles show data for PAMAM; squares show data for HDMBr; triangles show data for CDP.

In these experiments, we tested three NABPs (PAMAM-G3, HDMBr, and CDP) that differ in chemical composition but all can bind DNA effectively, with a dissociation constant in the range of 10^8^–10^9^ M^–1^ depending on the nature of the nucleic acid [22], [23]. These compounds were selected from a larger panel of polycations that can interact with nucleic acids both *in vivo* and *in vitro*, with studies suggesting an acceptable level of toxicity [23]. To determine first whether these compounds can influence anti-DNA binding, we tested a murine monoclonal antibody called QB1 that is specific for DNA. In these experiments, we added NABPs at various concentrations followed immediately by the monoclonal antibody. As data in Figure 1 show, all three compounds blocked the binding of QB1 to the DNA.

### Inhibition of Lupus Anti-DNA Binding to DNA by NABPs

While the compounds blocked QB1 binding, plasma can contain a much wider array of specificities that can differ in avidity and fine specificity in a way that could influence inhibition by NABPs [24]. To determine whether NABPs can inhibit anti-DNA produced in the setting of disease, we next tested human SLE plasma. For this purpose, we screened a panel of patient plasmas and selected three with the highest titers of anti-DNA levels for detailed analysis. Since levels of anti-DNA vary markedly during disease, only a limited number of plasmas had sufficient activity for this purpose.

**Figure 2:**
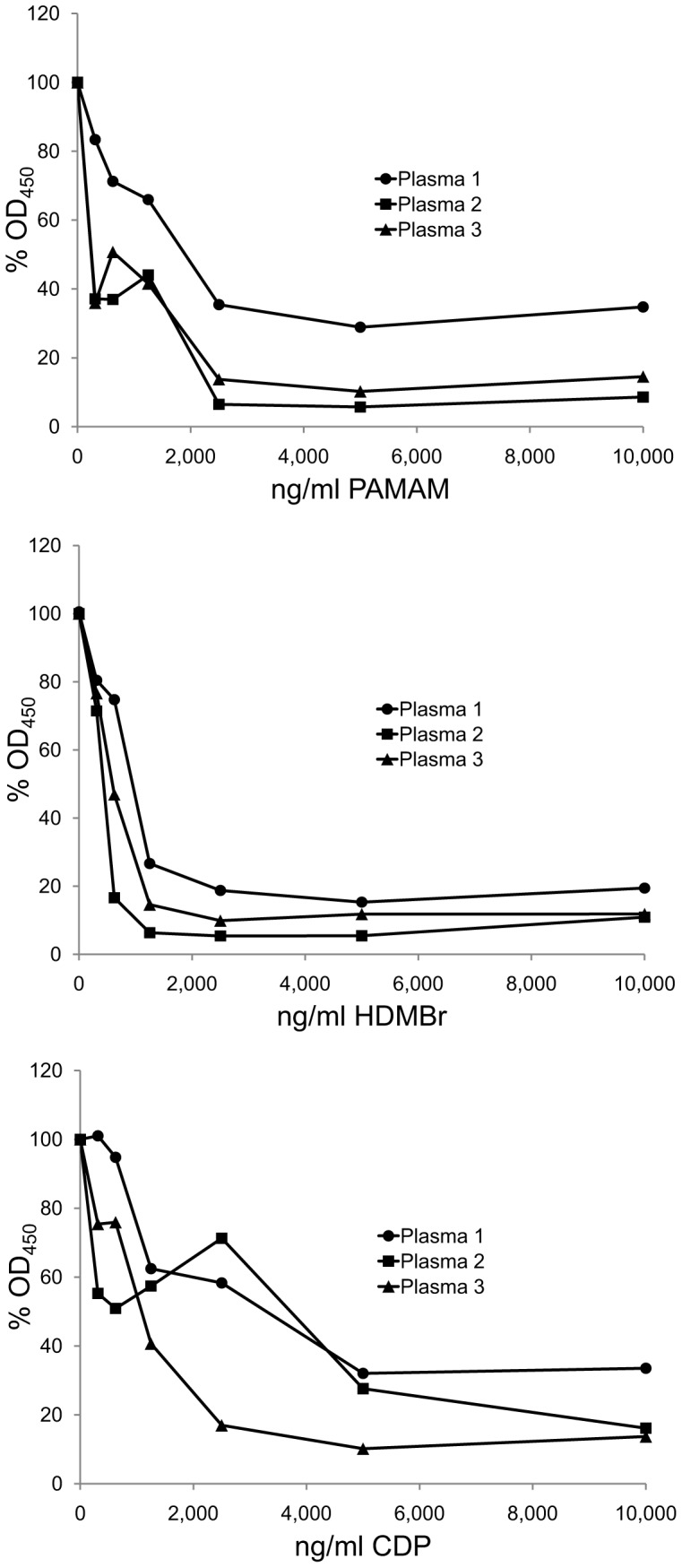
The inhibition of SLE antibodies to DNA by NABPs. The ability of polymers PAMAM, HDMBr, and CDP to inhibit DNA binding by anti-DNA antibodies in three SLE patient plasmas to DNA was tested by ELISA. Binding by antibodies in Plasma 1 (final dilution 1/1,000), Plasma 2 (final dilution 1/2,800), and Plasma 3 (final dilution 1/3,000) was assayed in the presence of PAMAM, HDMBr, or CDP at final concentrations ranging from 300 ng/ml to 10,000 ng/ml or with dilution buffer alone. Antibody levels were determined by ELISA as described in Materials and Methods. The OD_450_ values of 2 wells for each condition were averaged. Each point shown is 100% x average OD_450_ with polymer/average OD_450_ without polymer. Circles show data for Plasma 1; squares show data for Plasma 2; triangles show data for Plasma 3.

In the initial studies with human SLE plasmas, we tested the inhibitory activity of the NABPs in conventional ELISA assays in which native, double stranded (ds) DNA is bound directly to the surface of microtiter plates. As data in Figure 2 show, the three NABPs all inhibited anti-DNA binding over a wide range of concentrations. Similarly, these compounds also inhibited binding to single stranded (ss) DNA (data not shown).

### Specificity of NABP Inhibition

**Figure 3:**
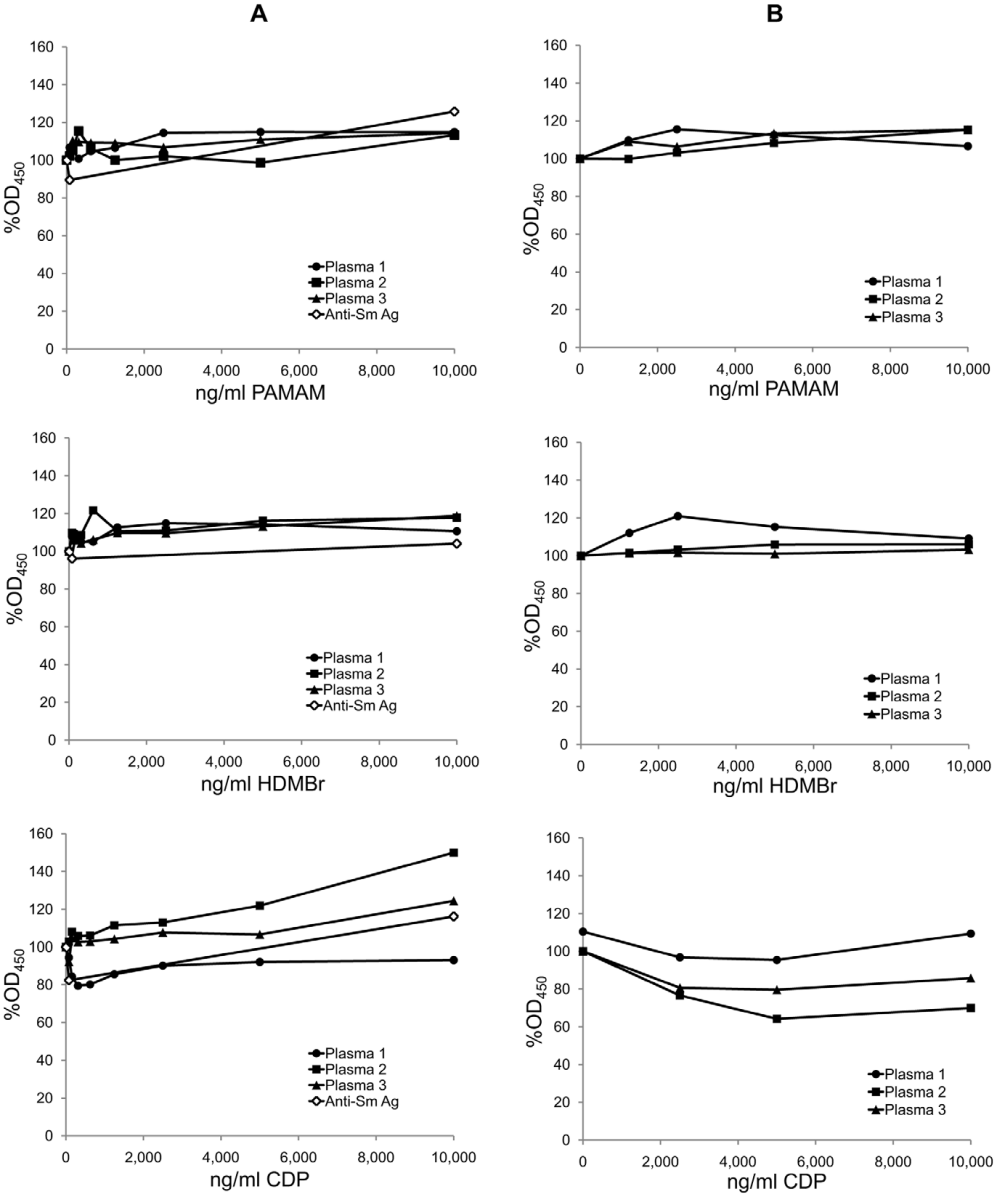
The effects of NABPs on the binding of SLE antibodies to the Sm antigen and tetanus toxoid. The specificity of inhibition of SLE antibody binding to dsDNA by NABPs was investigated by examining the effects of the polymers on SLE antibody binding to the Sm antigen and to tetanus toxoid in ELISA assays. (A) The effects of polymers PAMAM, HDMBr, and CDP on antibody binding to the Sm antigen were tested by ELISA using the three lupus plasmas and a human polyclonal anti-Sm antibody preparation (anti-Sm Ag) from USB. Plasmas were tested at 1/200 final dilution in the presence of inhibitors at concentrations from 80 ng/ml to 10,000 ng/ml of each polymer or with no polymer. The OD_450_ values of 2 wells for each condition were averaged. Each point shown is 100% x average OD_450_ with polymer/average OD_450_ without polymer. Circles show data for Plasma 1; squares show data for Plasma 2; triangles show data for Plasma 3; and open diamonds show data for the USB anti-Sm antibody. (B) The effects of polymers PAMAM, HDMBr, and CDP on antibody binding to tetanus toxoid were tested by ELISA using the three lupus plasmas. Plasma 1 (final dilution 1/720), Plasma 2 (final dilution 1/6,400), or Plasma 3 (final dilution 1/14,400) was incubated in wells coated with tetanus toxoid in the presence of PAMAM, HDMBr, or CDP at concentrations of 1,250 to 10,000 ng/ml or with no polymer. Antibody binding was determined as described in Materials and Methods. The OD_450_ values of 2 wells for each condition were averaged. Each point shown is 100% x average OD_450_ with polymer/average OD_450_ without polymer. Circles show data for Plasma 1; squares show data for Plasma 2; triangles show data for Plasma 3.

As a control for specificity, we tested the inhibitory capacity of these compounds using as antigens Sm (Smith antigen) and tetanus toxoid. The Sm antigen is a prominent lupus autoantigen that consists of proteins that can bind RNA; the epitopes bound by antibodies, however, are on the proteins and not the RNA [2]. As data in Figure 3 indicate, the compounds had only a limited effect on the antibody binding to Sm or tetanus toxoid when tested at concentrations as high as 10 µg/ml. These findings thus support the observation with the monoclonal antibody and lupus sera and indicate the specificity of NABPs inhibitors.

### Inhibition of Anti-DNA Binding by NABPs to DNA in Other Antigenic States

**Figure 4:**
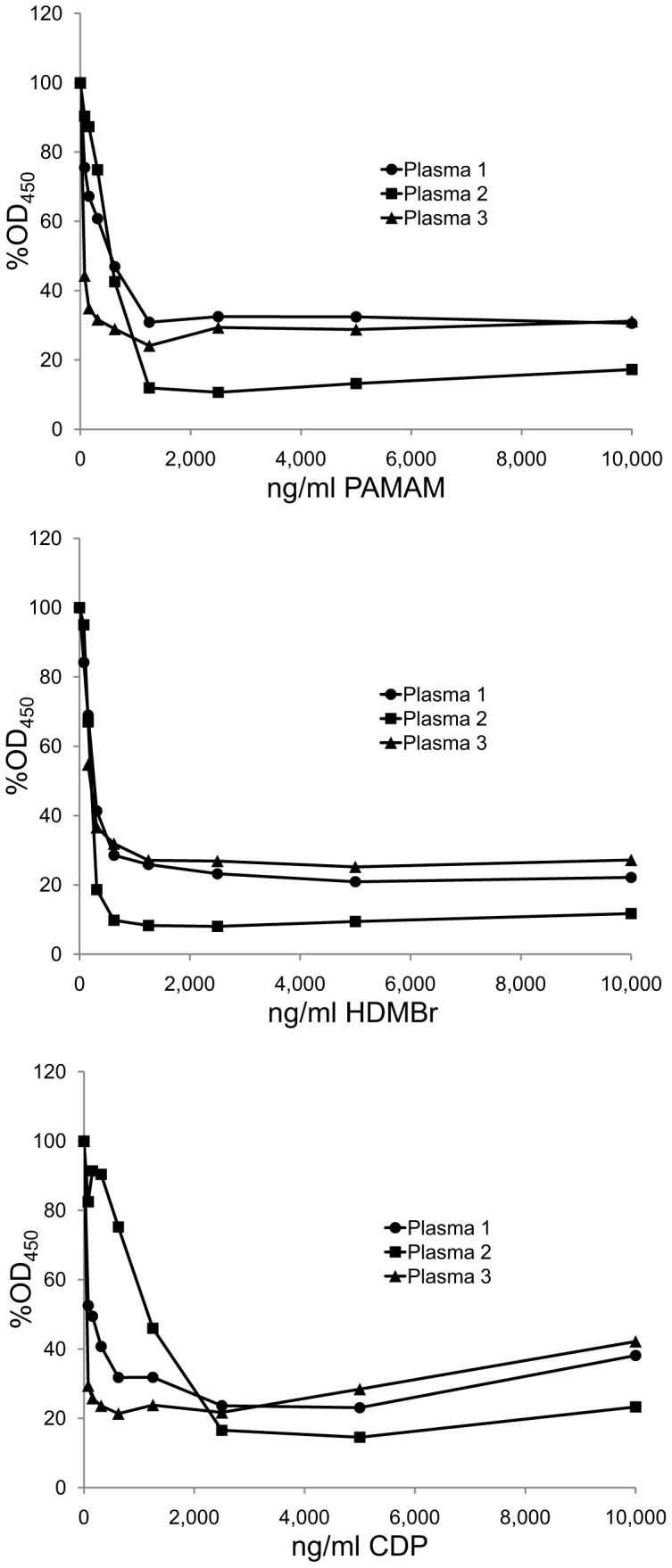
The effects of NABPs on the binding of SLE antibodies to biotinylated DNA. Native CT DNA was biotinylated and then captured in ELISA plate wells coated with streptavidin. The binding of Plasma 1 (final dilution 1/3,500), Plasma 2 (final dilution 1/8,000), and Plasma 3 (final dilution 1/4,500) was measured by ELISA in the presence of PAMAM, HDMBr, or CDP at final concentrations from 80 ng/ml to 10,000 ng/ml or with dilution buffer alone. The OD_450_ values of 2 wells for each condition were averaged. Each point shown is 100% x average OD_450_ with polymer/average OD_450_ without polymer. Circles show data for Plasma 1; squares show data for Plasma 2; triangles show data for Plasma 3.

The binding of antibodies to DNA that is directly adherent to plates can be influenced by surface effects and restriction on conformational adaptation of the immobilized DNA for antibody binding [25]. To provide a potentially more free or mobile antigenic state of DNA, we used biotinylated DNA antigen bound to plates coated with streptavidin. As shown previously, biotinylated DNA, depending on its size, shows much greater antigenicity than observed with direct binding of DNA to plates; this situation likely relates to a greater ability of DNA to undergo structural rearrangement necessary for antibody binding [25]. As shown in Figure 4, with this ELISA format as well as the direct ELISA, the NABPs blocked anti-DNA binding with similar dose-response curves.

### Displacement of Anti-DNA from Pre-formed Complexes by NABPs

**Figure 5:**
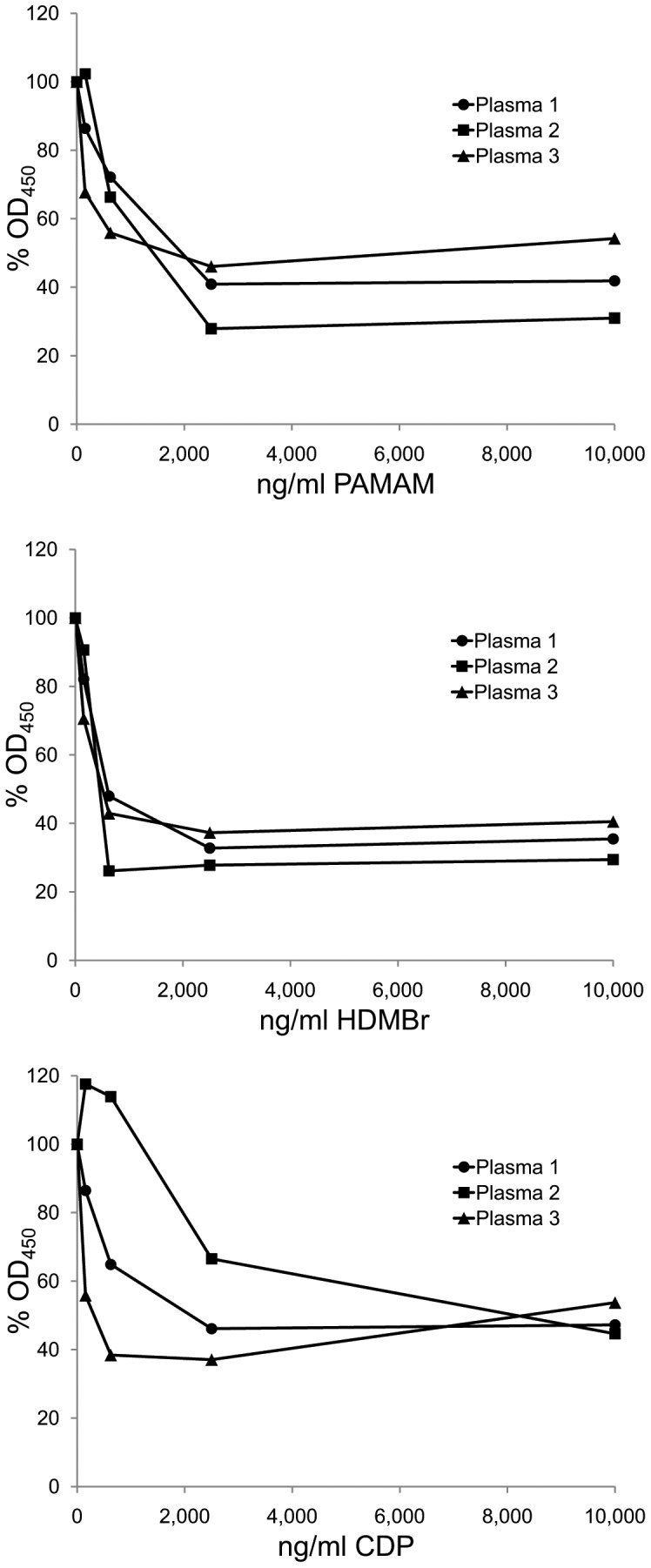
The effects of NABPs on the pre-formed complexes. In this assay, 50 µl/well of Plasma 1 diluted 1/1,700, Plasma 2 diluted 1/3,950, and Plasma 3 diluted 1/2,250 were incubated in wells of microtiter plates with biotinylated DNA bound to streptavidin. After 1 hour to allow the formation of immune complexes, 50 µl of dilutions of PAMAM, HDMBr, or CDP or 50 µl of dilution buffer alone were then added to produce concentrations of the polymers of 10,000 ng/ml, 2,500 ng/ml, 620 ng/ml, 160 ng/ml or 0 ng/ml. Antibody binding was then determined by ELISA. The OD_450_ values of 2 wells for each condition were averaged. Each point shown is 100% x average OD_450_ with polymer/average OD_450_ without polymer. Circles show data for Plasma 1; squares show data for Plasma 2; triangles show data for Plasma 3.

In the context of therapy with an NABP, some of the anti-DNA may be already bound to DNA in the form of an immune complex which represents the active state for pathogenicity. To determine whether NABPs can dissociate pre-formed immune complexes, we incubated biotinylated DNA with plasma for 1 hour to allow immune complexes to form. At that time point, the inhibitors were added. As the data in Figure 5 show, the NABPs significantly reduced antibody binding. These findings indicate that the NABPs can cause dissociation of pre-formed immune complexes, suggesting the utility of these NABPs to influence both the assembly and disassembly of the immune complexes that can serve as mediators in immunopathogenesis.

## Discussion

Results presented herein demonstrate that nucleic acid binding polymers can block, in a dose-dependent way, DNA-anti-DNA interactions in *in vitro* assays. These inhibitory activities occurred with native DNA and were observed with DNA bound to microtiter plates either directly or through attachment of biotinylated DNA to streptavidin. The attachment of DNA via biotin-steptavidin provides an antigenic form that more closely resembles the properties of DNA in solution than that of plate-bound DNA [25]. Furthermore, the NABPs could cause the dissociation of preformed DNA-anti-DNA immune complexes. As such, these findings suggest a new approach to the therapy of SLE based on the specific reduction of pathogenic immune complexes comprised of DNA and anti-DNA.

In these studies, we have focused attention on three representative NABPs. PAMAM-G3 is a third-generation dendrimer comprised of branching polyamidoamine structures with a high density of primary amino groups on the surface. This polymer has been widely used for drug and gene delivery [26], [27]. HDMBr or polybrene is a polycation that can bind DNA and has been used to promote DNA transfection into cells with either free DNA or viral vectors [28], [29]. Like other polycations, HDMBr can readily condense DNA into nanoparticles for intracellular delivery. Finally, CDPs are polymeric β-cyclodextrin-based structures typically synthesized by the condensation of a diamino-cyclodextrin with a diimidate [30]–[34]. A wide range of physicochemical properties with respect to charge density, molecular weight, backbone rigidity and hydrophobicity has been obtained for CDP [30]–[34]. The cationic CDP used in this study has been evaluated in a Phase I clinical trial for siRNA delivery [35].

The range of polymers that can bind DNA is extensive and, depending on intended use, NABPs can be mixed with other molecular entities to provide properties suited for vector function. These properties include stable interaction with DNA and protection against nucleases prior to interaction with cells [36]. For gene delivery, however, the interaction with DNA cannot be too strong since DNA must be released from the vector to allow transfection. Gene therapy applications of NABPs impose distinct structural requirements on polymers related to their function and desired properties in an intracellular vs. extracellular location [37]. In the setting of lupus, however, the use of NABPs to block anti-DNA binding will involve the extracellular space and can involve concentrations of DNA and NABP much lower than those used to create a vector. Indeed, our results indicate that these compounds can act at low concentrations, likely reflecting their avidity of DNA binding [38].

Our experiments demonstrated that, in addition to blocking the binding of anti-DNA to DNA, NABPs can dissociate immune complexes formed with DNA antigen. This inhibition occurred in the presence of antibody following the addition of the inhibitors. There are at least two explanations for this activity. First, the presence of the NABPs could induce conformational changes in the DNA antigen, reducing the avidity of antibody binding and causing dissociation [39]. In this case, by binding DNA, the NABPs could distort the backbone structure of the DNA and reduce antigenicity.

An alternative explanation for this finding relates to the mode of interaction of anti-DNA with DNA. As shown in prior studies, many anti-DNA antibodies bind to DNA by a mechanism termed monogamous or bivalent interaction [40]. According to this model, stable binding requires simultaneous interaction of each combining site with an antigenic determinant along the same extended DNA molecule. The size of this DNA piece corresponds to a stretch of about 40 nucleotides which is far greater than the number needed to fill an antibody combining site that can accommodate up to 6 bases. The large piece is needed to allow intramolecular cross-linking and sufficient avidity for a stable interaction.

Since its avidity for DNA is low, each combining site may undergo frequent on-off reactions, with stable interaction occurring because the antigenic sites are contiguous on the same molecule. As a consequence, the on-rate is increased because of the proximity of the antigen whose presence (and, effectively, high local concentration) is maintained because of the action of the other Fab combining site. In this situation, an NABP can compete for occupancy on the DNA with the combining site in the off position. The ability of NABPs to disassemble immune complexes would be important for blocking renal deposition as well as cytokine stimulation.

In this regard, we have also tested the ability of the polymers to inhibit anti-DNA binding in the *Crithidia luciliae* assay; this assay tests binding by immunofluorescence to the kinetoplast of *Crithidia* organisms (a parasite of the blowfly) which have been fixed to a slide. Of the three plasmas we tested by ELISA, plasmas 1 and 2, but not 3, showed significant binding in this assay; differences in binding of anti-DNA to various antigen substrates is well known and likely relates to specificity and avidity differences among antibodies detected with different DNA antigens. Nevertheless, with plasmas 1 and 2, we observed that HDMR but not the other polymers inhibited binding as assessed by immunofluorescence. These observations support findings with the ELISA and suggest that the range of polymers that may inhibit anti-DNA binding may vary depending on DNA substrate (Stearns and Pisetsky, preliminary data).

In our studies, we focused attention on three plasmas that displayed significant amounts of anti-DNA activity although we have confirmed these results with other plasmas that also had appreciable anti-DNA activity [Stearns and Pisetsky, preliminary data]. In view of data indicating that antibodies to DNA show overall similarity to interaction with the DNA backbone [3], [4], the inhibition of anti-DNA binding by NABPs may be a general phenomenon that can form of the basis of new therapies in lupus to both block nephritis and attenuate cytokine disturbances. Studies are therefore in progress to test this possibility both *in vitro* and *in vivo* in animal model systems.

## Materials and Methods

### Antibodies and Plasma

The murine monoclonal antibody QB1 was a gift of Dr. Marc Monestier of Temple University. This antibody was derived from an A.SW mouse treated with quinidine [41].This antibody binds double stranded DNA. Plasmas (denoted as 1–3) from patients with systemic lupus erythematosus were purchased from Plasma Services Group (Southhampton, PA) and were selected from among a panel for high binding to DNA by an ELISA. A polyclonal human anti-human Sm (Smith) antigen antibody (USB anti-Sm) was from US Biological (Swampscott, MA).

### Polymers

PAMAM-G3 (polyaminamine dendrimer, 1,4-diamino butane core, generation 3.0) and HDMBr (MW 4000-6000) were purchased from Sigma Chemical Company (St. Louis, MO). CDP was kindly provided by Dr. Mark Davis (California Institute of Technology, Pasadena, CA) [30], [33].

### Anti-DNA ELISA

Antibodies to DNA were assayed by ELISA with DNA directly bound to microtiter plates or with biotinylated DNA bound to microtiter plates coated with streptavidin. These experiments used Immulon 2HB (high binding) flat-bottom, 96 well microtiter plates (Thermo Scientific,Waltham, MA). Unless otherwise noted, samples had a final volume of 100 µl/well, with incubations at room temperature (21–23°C) for 1 h. Washes used phosphate buffered saline (PBS) at room temperature while blocking used 200 µl/well of 0.5% bovine serum albumin, 0.05% Tween 20 in PBS for 2 h at room temperature. In all experiments, plasma or antibodies were diluted in buffer consisting of 0.1% bovine serum albumin and 0.05% Tween 20 in PBS.

At the end of the incubation with antibody or plasma, wells were washed, and then incubated with a 1/1,000 dilution of either anti-mouse IgG (whole molecule) peroxidase-conjugated antibody (Sigma Chemical Company, St. Louis, MO) or anti-human IgG (gamma-chain specific) peroxidase-conjugated antibody (Sigma) in ELISA dilution buffer. The final step was incubation with 100 µl/well of horseradish peroxidase substrate (0.015% 3,3′,5,5′-tetramethylbenzidine dihydrochloride, 0.01% H_2_O_2_ in 0.1 M citrate buffer, pH 4) for 30 min at room temperature. The peroxidase reaction was stopped by adding 100 µl of 2 M H_2_SO_4_ to each well, and the absorbance of each well was read at 450 nm with an UVmax spectrophotometer. For the direct binding assay, wells of ELISA plates were coated with 5 µg/ml native double stranded calf thymus (CT) DNA (Worthington Biochemical, Lakewood, NJ) in 1 x SSC (150 mM NaCl, 15 mM Na citrate, pH 7) overnight at 4°C. Wells were washed, and then blocked. For assays involving biotinylated DNA, native CT DNA was purified by phenol:chloroform extraction and then diluted to 0.5 mg/ml in 10 mM Tris, 1 mM EDTA buffer pH 8.0 (TE buffer). Photoprobe® (Long Arm) Biotin, citrate salt (Vector Laboratories, Burlingame, CA) was diluted to 1 mg/ml with sterile, distilled water. Forty microliters of each solution were combined in a 16×100 mm glass tube. The tube was placed on ice and biotinylation was performed by irradiation with a Blak-Ray UV lamp without a filter, positioned 7.5 cm from the open end of the tube. Following butanol extraction, DNA was precipitated with ethanol and collected by centrifugation. The pellet of biotinylated DNA was resuspended in TE buffer [25]. For the ELISA with biotinylated DNA, wells were first coated with 2 µg/ml streptavidin (Roche Applied Science, Indianapolis, IN) in sodium phosphate buffer pH 9.0 overnight at 4°C. Wells were washed, and then blocked. Wells were then incubated with 1 µg/ml biotinylated DNA overnight at 4°C. Following washing, the ELISA was performed as described for the direct binding assay. To determine whether NABPs could displace antibody from pre-formed complexes, biotinylated DNA was used as the antigen. Fifty microliters of SLE Plasmas 1–3 (diluted 1/1,700, 1/3,950 and 1/2,250 respectively) were incubated with DNA coated plates for 1 h at room temperature to allow immune complexes to form. At that time, 50 µl of polymers (0.3 −20 µg/ml) were added for 1 h at room temperature. Wells were washed, and antibody binding was determined as described.

For inhibition assays, concentrations of the QB1 antibody and dilutions of human SLE plasmas were determined by prior titration to provide an OD_450_ of approximately 1. Anti-DNA QB1 antibody was used at a final concentration of 35 ng/ml.

### Anti-Sm Inhibition ELISA

Wells of ELISA plates were coated with 100 µl Sm (Smith) antigen from calf thymus (United States Biological, Swampscott, MA) diluted in 0.1 M sodium phosphate buffer pH 9.0, washed and then blocked. Dilutions of SLE patient plasmas and a commercial polyclonal human anti-Smith antigen antibody (United States Biological, Swampscott, MA, catalog number S1014-25A) were then added to wells with or without polymer in a final volume of 100 µl. Antibody binding was determined as described above. SLE Plasmas 1–3 were used at a final dilution of 1/200. The USB anti-Sm was used at a final dilution of 1/200.

### Anti-tetanus Toxoid Inhibition ELISA

Wells of ELISA plates were coated with 100 µl of 1 µg/ml purified tetanus toxoid (gift from Richard M. Scearce, Duke University Medical Center, Durham, NC) in 0.1 M sodium phosphate buffer pH 9.0. This ELISA was conducted as described for the anti-Sm ELISA above, except that the SLE Plasma 1, 2 and 3 dilutions were 1/720, 1/6,400, and 1/14,400, respectively.
